# Predicting Social Frailty in Older Adults Using Fitbit-Derived Circadian and Heart Rate Biomarkers: Cross-Sectional Study

**DOI:** 10.2196/71393

**Published:** 2025-07-24

**Authors:** Hiroki Maekawa, Yu Kume

**Affiliations:** 1 Department of Rehabilitation Faculty of Health Sciences Tohoku Fukushi University Sendai Japan; 2 Doctoral Course of Health Sciences Graduate School of Medicine Akita University Akita Japan; 3 Department of Health Sciences Graduate School of Medicine Akita University Akita Japan

**Keywords:** social frailty, digital biomarkers, Fitbit, rest-activity rhythm, heart rate metrics, nonparametric indexes, extended cosinor model, community-dwelling older adults

## Abstract

**Background:**

Social frailty poses a potential risk even for relatively healthy older adults, necessitating development of early detection and prevention strategies. Recently, consumer-grade wearable devices have attracted attention due to their ability to continuously collect physiological and activity-related data. These data can potentially be used to calculate digital biomarkers for screening social frailty in older adults.

**Objective:**

The objective of this study was to explore digital biomarkers associated with social frailty using sensor data recorded via Fitbit devices and evaluate their relationship with health outcomes in older adults.

**Methods:**

This cross-sectional study was conducted in 102 community-dwelling older adults. Participants attending frailty prevention programs wore devices from the Fitbit Inspire series on their nondominant wrist for at least 7 consecutive days, during which step count and heart rate data were collected. Standardized questionnaires were used to assess physical functions, cognitive functions, and social frailty, and based on the scores, the participants were categorized into 3 groups: robust, social prefrailty, and social frailty. The sensor data were analyzed to calculate nonparametric and extended cosinor rhythm metrics, along with heart rate–related metrics.

**Results:**

The final sample included 86 participants who were categorized as robust (n=28, 33%), social prefrailty (n=39, 45%), and social frailty (n=19, 22%). The mean age of the participants was 77.14 (SD 5.70) years, and 91% (78/86) were women. Multinomial logistic regression analysis revealed that a step-based rhythm metric (intradaily coefficient of variation) was significantly associated with social frailty (odds ratio 1.05, 95% CI 1.01-1.11; *P*=.01). The heart rate metrics, including the delta resting heart rate and time of transition from rest to activity, showed significant associations with both social prefrailty (odds ratio 0.82, 95% CI 0.68-0.99; *P*=.04) and social frailty (odds ratio 0.69, 95% CI 0.50-0.95; *P*=.01). Specifically, delta resting heart rate, defined as the difference between the overall average heart rate and resting heart rate, exhibited significant negative associations with social prefrailty (odds ratio 0.82, 95% CI 0.68-0.97; *P*=.02) and social frailty (odds ratio 0.74, 95% CI 0.58-0.94; *P*=.02). Furthermore, analysis using a linear regression model revealed a significant association between the intradaily coefficient of variation and the word list memory score, a measure of cognitive decline (β=−0.04; *P*=.02).

**Conclusions:**

This study identified associations between novel rhythm and heart rate metrics calculated from the step count and heart rate recorded by Fitbit devices and social frailty. These findings suggest that consumer-grade wearable devices, which are low cost and accessible, hold promise as tools for evaluating social frailty and its risk factors through enabling the calculation of digital biomarkers. Future research should include larger sample sizes and focus on the clinical applications of these findings.

## Introduction

### Background

In an aging society, early detection and prevention of frailty are urgent priorities. Frailty is a multifaceted concept encompassing physical, cognitive, and social aspects, all of which play critical roles in comprehensively assessing the health status of older adults [1,2]. Among these, social frailty precedes physical frailty and has been reported to promote unhealthy aging while increasing the risks of all-cause mortality and functional impairment [3,4]. Bunt et al [5] proposed that, in addition to general resource–related factors such as living alone and economic difficulties, social resource factors such as connections with neighbors and friends, as well as basic social needs such as social behavior, social isolation, and social support, are critical components of social frailty. Social frailty profoundly affects the lifestyles of older adults and has been associated with a poor nutritional status, depression, reduced physical capability, and decreased activity levels [6]. Furthermore, it has been linked to declines in activities of daily living [7] and instrumental activities of daily living [8,9]. Rest-activity rhythm (RAR) has long garnered attention as an established indicator for evaluating health conditions related to such lifestyle factors.

Previous studies using actigraphy have used RAR parameters derived from activity trackers to predict various health outcomes, including physical frailty, cognitive decline, and depression [10-14]. These parameters quantify 24-hour cycles of rest and activity based on activity counts and have been widely used in health research. However, their application to social frailty remains underexplored [15], and studies leveraging wearable sensors for this purpose are particularly scarce.

Recently, wearable devices such as Fitbit smartwatches have enabled the collection of detailed physiological and behavioral data, including heart rate, respiratory rate, and sleep data in addition to traditional activity data. These advancements have spurred the development of innovative digital biomarkers such as heart rate–based rhythm metrics, which complement conventional activity count–based indicators [16,17]. Fitbit devices, known for their affordability and accessibility, have demonstrated accuracy comparable to research-grade devices for measuring step count and heart rate [18-20]. Despite their potential, the use of wearable devices to evaluate social frailty and its associated risk factors remains limited, highlighting the need for further investigation, such as studies similar to the one reported in this paper.

### Objectives

Fitbit and other wearable devices provide step count, heart rate, and other data that allow for the calculation of digital biomarkers to predict health outcomes. However, studies using raw data from these devices to compute novel metrics and evaluate their associations with health outcomes remain insufficient.

The primary aim of this study was to identify digital biomarkers associated with social frailty. Social frailty has been linked to reduced daytime physical activity, as demonstrated in previous research [7,9]. Although there is limited evidence directly connecting social frailty to heart rate, it is plausible that decreased physical activity in individuals with social frailty leads to lower overall heart rate variability (HRV) and altered heart rate patterns. Therefore, we hypothesized that the frailty group would exhibit significant associations with RAR parameters (nonparametric and extended cosinor analysis) and heart rate–based parameters (eg, nighttime heart rate and resting heart rate [RHR]), assessed by leveraging Fitbit data. These parameters have the potential to shed light on the physiological and behavioral characteristics of social frailty.

The secondary aim of this study was to explore the relationship between RAR metrics derived from Fitbit data and cognitive function. While previous research has linked nonparametric and cosinor-based RAR metrics to cognitive decline, most studies have relied on research-grade actigraphy devices. This study introduces novel RAR metrics derived from Fitbit data to exploratorily assess their associations with cognitive function.

## Methods

### Study Design and Participants

This study was designed as a cross-sectional analysis to investigate the relationship between digital biomarkers and social frailty in community-dwelling older adults in Akita Prefecture, Japan. To determine the appropriate sample size for the logistic regression analysis, we used G*Power (version 3.1.9.6) with the likelihood-ratio test as the test statistic. The estimation indicated that a minimum of 82 participants would be required to detect a clinically significant effect based on the following parameters: 3 groups, a significance level (α) of .05, a statistical power of 80%, and an odds ratio (OR) of 2.0 [21]. Considering a potential dropout rate of 20% to 30%, we recruited a total of 102 older adults between October 2023 and December 2024. Recruitment sites included municipally organized programs conducted at care management centers in the Yabase, Kawabe, Ushijima, Goshono, Iijima, and Shimoshinjo districts of the city of Akita, as well as in the city of Yurihonjō and the town of Mitane. Additional participants were recruited from a community-based exercise group regularly followed by the principal investigator held at a temple in the Katsuhira area. The inclusion criteria were (1) age of ≥65 years, (2) independent walking ability in daily life, and (3) residence at home. The exclusion criteria were (1) a central nervous system disorder, (2) requiring assistance in daily living, (3) a cardiac condition, or (4) requiring support or care as certified under Japan’s public long-term care insurance system.

Data collection used consumer-grade fitness trackers, specifically the Fitbit Inspire 2 and 3 (Google). The accuracy of Fitbit data has been evaluated in several studies. According to systematic reviews [22,23], Fitbit devices consistently demonstrate acceptable levels of accuracy for daily step counts and heart rate measurements despite a tendency to underestimate these metrics under certain conditions. Generally, Fitbit wearables provide activity assessment accuracy comparable to research-grade devices but tend to overestimate moderate to vigorous physical activity under free-living conditions. One of the major challenges faced by medical researchers when interpreting physical activity data is related to participants forgetting to wear the activity tracker [23]. However, the Inspire series of Fitbit devices, being waterproof and offering a battery life of up to 10 days, minimizes interruptions in data collection. These features contribute to ensuring high-quality data with fewer missing values, making them particularly advantageous for calculating rhythm metrics.

Participants were instructed to wear the device for at least 7 consecutive days. The research staff placed the device on the participants’ nondominant wrist, approximately 1 inch below the wrist bone, ensuring that the back of the device was in contact with the skin. Participants were instructed to wear the tracker at all times, including during bathing, whenever possible. However, they were advised not to use the device in situations prohibited by the manufacturer, such as diving under high water pressure or in high-temperature environments such as saunas.

### Ethical Considerations

This study was conducted in accordance with the ethical principles outlined in the Declaration of Helsinki. The research protocol and informed consent forms were approved by the institutional review board of Akita University School of Medicine (approval 3063). All participants provided written informed consent before taking part. This study involved both the collection of wearable device data and in-person assessments, including evaluations of social frailty and cognitive performance. To ensure participant confidentiality and data protection, all collected data were anonymized before analysis. Participants did not receive monetary compensation, nor did they incur any costs by taking part in the study. As a token of appreciation, each participant received a booklet summarizing their individual results and interpretations.

### Classification of Social Frailty Index and Assessment

Demographic data, including age (years), sex (female or male), and educational level (years), were collected from all participants. Social frailty was classified using the social frailty index developed by Makizako et al [24], which reflects the accumulation of social risk factors and indicates reduced social participation or roles. The index consists of five components: (1) living alone (yes), (2) talking with someone every day (no), (3) feeling helpful to friends or family (no), (4) going out less frequently compared with the previous year (yes), and (5) visiting friends sometimes (no). Participants with a total score of 0 were classified as robust, those with a score of 1 were classified as social prefrailty, and those with scores of 2 to 5 were classified as social frailty. Physical performance was assessed by measuring grip strength (kg) and usual walking speed (m/s) over a 5-m course. Cognitive performance was evaluated using 4 subtests from the National Center for Geriatrics and Gerontology–Functional Assessment Tool [25]. The word list memory (WLM) test included immediate recognition and delayed recall tasks, where the mean correct answers across 3 trials (range 0-10) and the number of correct answers in delayed recall (range 0-10) were summed to calculate a total score (range 0-20). The trail making test versions A and B was used to measure cognitive flexibility, with completion times recorded in seconds. Finally, the digit symbol substitution task was conducted to assess information processing speed, where the number of correct responses in 90 seconds was recorded.

### Wearable Data Preprocessing

After the monitoring period, devices were collected, and their stored data were synchronized via Bluetooth to a smartphone before being transferred to the Fitbit database. Researchers then accessed the Fitbit developer platform linked to a Google account [26], generated an access token (refreshed every 8 hours), and executed a Python script (Python Software Foundation) in Google Colaboratory to retrieve the raw step count and heart rate data at 1‑minute intervals. To ensure data integrity, periods without heart rate data were excluded, and corresponding step count data were marked as invalid; only time points with both valid heart rate and step count data were retained as clean data. Consistent with the work by Rykov et al [17], a valid day was defined as one with at least 20 hours of concurrent heart rate and step count data. On the basis of evidence that 5 days of wear yield reliable RAR parameters in older adults [27], only participants with at least 5 valid days were included in the study.

### Extraction of Digital Biomarkers

Raw data from Fitbit were used to extract digital biomarkers related to circadian rhythms and heart rate variation. Step count and heart rate data were processed using a modified version of the script developed by Rykov et al [17] to calculate both extended cosine-based and nonparametric circadian rhythm indexes.

#### The Extended Cosinor Metrics

Extended cosine-based indexes included the midline estimating statistic of rhythm (MESOR), acrophase, amplitude, minimum, pseudo–*F* statistic, time of transition from rest to activity (UpMesor), time of transition from activity to rest (DownMesor), and the α and β parameters. These indexes were derived using an extended cosinor model, which incorporates nonlinear transformations to refine the classic cosine function *c*(*t*):

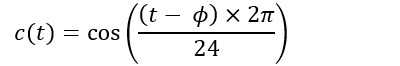



*r*(*t*) = mes + amp × *c*(*t*)

In these equations, *mes* is the rhythm-adjusted mean level, *amp* is the amplitude, and ϕ (acrophase) is the time of the mathematically defined peak.

In contrast, the extended cosinor model applies nonlinear transformations to *c*(*t*), enabling it to represent more complex waveform shapes. Three transformation methods are commonly used: (1) hill transformation, (2) antilogistic transformation, and (3) arctangent transformation.

These transformations allow for better modeling of circadian rhythms that deviate from simple cosine waveforms [28,29]. This study used the antilogistic transformation, which is defined as follows:

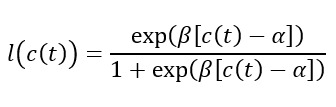



The extended cosinor model, incorporating this transformation, is expressed as *r*(*t*) = min + amp × *l*(*c*[*t*]), where *min* is the minimum value of the function, *amp* is the difference between the minimum and maximum values, *c*(*t*) represents the classic cosine wave, and *l*(*c*[*t*]) is the antilogistically transformed function.

Interpretation-wise, *min* is the minimum value of the function, *amp* is the difference between the minimum and maximum of the function, ϕ is the time at which *r*(*t*) has its mathematically well-defined “peak,” α controls the “width” of the function, and β controls the “steepness” of the function.

In this study, we used the extended cosinor model to analyze rhythmic patterns derived from Fitbit data. *Minimum* represents the lowest value of the fitted function, indicating the period of least activity or lowest heart rate within a 24-hour cycle. *Amplitude* measures half the extent of predictable variation within a cycle, serving as an indicator of the maximum achievable activity level. The α parameter determines whether the peaks of the curve are wider or narrower than the troughs; specifically, a small α value signifies narrow troughs and wide peaks, whereas a large α value indicates wide troughs and narrow peaks. The β parameter assesses the steepness of the rise and fall of the curve relative to a standard cosine wave, with large β values producing nearly square waveforms that represent abrupt transitions between high and low activity levels. *Acrophase* refers to the time of day at which the peak of the rhythmic pattern occurs, initially measured in radians and subsequently converted into time units (hours) and referred to as acrotime when necessary. *F_pseudo* measures the improvement in the fit obtained through the nonlinear estimation of the transformed cosine model, serving as an indicator of the robustness of the rhythm. *UpMesor* denotes the time of day when activity transitions from low to high, reflecting the timing of the RAR; lower (earlier) values suggest an earlier onset of daytime activity and a more advanced circadian phase. *DownMesor* indicates the time of day when activity transitions from high to low, representing the cessation of peak activity within the RAR; lower (earlier) values imply an earlier decline in daily activities, also suggesting a more advanced circadian phase.

**Figure 1:**
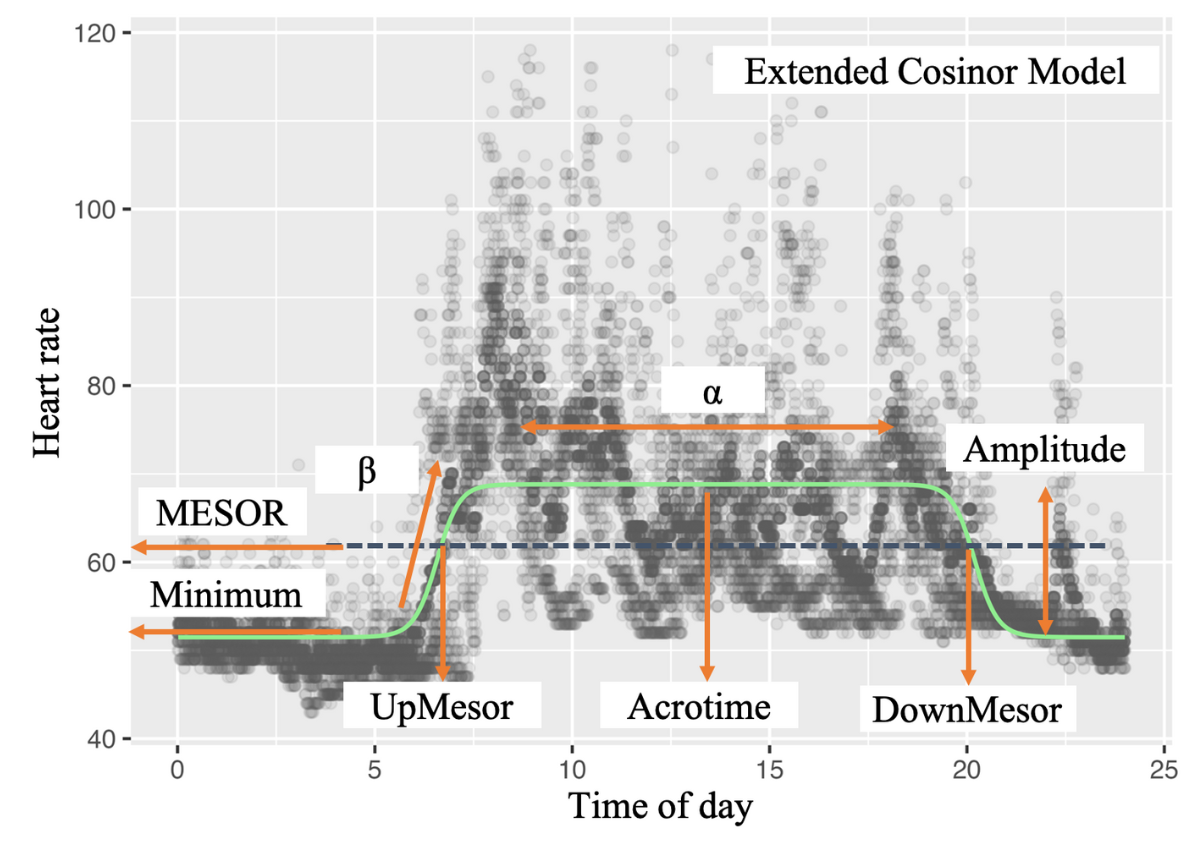
Illustration of the extended cosinor model applied to 24-hour heart rate data from a representative participant. The green curve represents the fitted extended cosinor function used to estimate rhythm-related parameters: minimum (lowest value), amplitude (half the predictable variation), midline estimating statistic of rhythm (MESOR; 24-hour mean), acrotime (time of peak), transition from rest to activity (UpMesor), transition from activity to rest (DownMesor), α (width of peak), and β (steepness of transition). Data were collected as part of a cross-sectional study conducted between October 2023 and December 2024 using Fitbit Inspire 2 and 3 devices worn by community-dwelling older adults in Akita Prefecture, Japan.

Finally, MESOR is an estimated 24-hour average activity level calculated as the sum of the minimum plus half the amplitude (MESOR = [minimum + amplitude]/2) and provides a measure of the central tendency in the daily activity cycle. It should be noted that the MESOR in the extended cosinor model differs from the MESOR in the standard cosinor model [29]. A visual representation of the extended cosinor variables used in this study is presented in Figure 1.

Compared to the classic cosine model, the extended cosinor model provides greater flexibility to capture complex waveform shapes. The extended cosinor model—through the application of an antilogistic transformation—can accommodate, for example, rapid activity onset in the morning, sustained daytime activity, and gradual transitions into nighttime rest. This approach enables a more realistic fit of circadian patterns while preserving interpretability [29].

#### Nonparametric Metrics

Nonparametric indexes included interdaily stability (IS), intradaily variability (IV), daytime activity level (most active 10-hour activity level [M10]), nighttime activity level (least active 5-hour activity level [L5]), relative amplitude (RA), intradaily coefficient of variation (ICV), autocorrelation, and peaks. Among these, IS, IV, M10, L5, and RA were originally proposed in studies by Van Someren et al [30] and Witting et al [31]. ICV, autocorrelation, and peaks have been introduced in more recent studies to provide complementary insights into circadian rhythm variability and stability.

IS measures the stability and regularity of activity patterns across 24-hour cycles. It is calculated as the ratio of the variance of the average 24-hour activity profile to the total variance of data aggregated by the hour across all days. A higher IS value indicates a more stable and regular circadian rhythm.

IV quantifies the fragmentation of rest and activity periods within a 24-hour cycle. It is calculated as the mean square of the differences between successive hourly aggregated data normalized by the total variance across all days. A higher IV value indicates a more fragmented rhythm, characterized by shorter alternating periods of rest and activity rather than 1 extended active period and 1 extended rest period.

RA reflects the difference between the most and the least active periods during the day. It is calculated as the difference between M10 (mean activity level during the most active 10 hours of the day) and L5 (mean activity level during the least active 5 hours of the day) divided by the sum of M10 and L5. Higher RA values indicate a greater amplitude in activity levels.

ICV is a novel measure of rhythm stability proposed by Rykov et al [17]. Unlike IS, which evaluates the stability and regularity of daily rhythms across days by calculating the variance of the mean 24-hour activity profile relative to the total variance, ICV focuses on the variation within a single day. ICV is calculated as the 24-hour mean of the coefficients of variation (CVs), where the CV is the ratio of the SD of the mean for each hour across days. Higher ICV values indicate greater variation and less stable rhythms within a day. In general, individuals with more regular and healthy lifestyles tend to exhibit higher IS values, reflecting their stable and consistent daily rhythms, and lower ICV values, indicating more stable activity patterns within each hour of a day. Conversely, higher ICV values may suggest fragmented or inconsistent activity patterns, often characterized by intermittent bursts of activity and prolonged periods of inactivity.

Autocorrelation is another measure of rhythm stability calculated as the lagged autocorrelation of time-series data. Autocorrelation was computed for time series aggregated into 15-, 30-, and 60-minute intervals, with a lag corresponding to 1 day.

In addition, a robust peak detection algorithm based on *z* scores was applied to the time-series data to identify peaks in steps and heart rate data [32]. For this, the daily mean number of peaks and its SD were calculated.

#### Heart Rate Metrics

Extracted heart rate–based metrics included the overall average heart rate, RHR, delta RHR (dRHR), daytime and nighttime heart rate, and the variability of these measures calculated using the SD and CV. In addition, the root mean square of successive differences (RMSSD) of the heart rate was calculated.

RHR was defined as the average heart rate during 15-minute intervals with 0 recorded steps, representing resting periods. Daytime heart rate was calculated as the mean heart rate between 2 PM and 4 PM, whereas nighttime heart rate was calculated by averaging values from 3 consecutive 2-hour intervals: midnight to 2 AM, 2 AM to 4 AM, and 4 AM to 6 AM. These intervals and metrics were selected following the approach used by Rykov et al [17].

The SD and CV were computed for each heart rate measure to assess variability. dRHR, reflecting the difference between activity and rest, was calculated as the difference between the overall average heart rate and RHR. RMSSD, an established metric for assessing HRV, was calculated using both raw data and hourly aggregated data.

#### Custom Modifications to Digital Biomarker Calculation Programs

The calculation of digital biomarkers in this study was conducted using step and heart rate data by executing the R script provided by Rykov et al in R (version 4.4.1; R Foundation for Statistical Computing). However, for extended cosinor–based metrics, the *ActCR* package available on the Comprehensive R Archive Network was used instead of the actigraphy package used by Rykov et al [17]. The *ActCR* package offers functionality to calculate a greater number of extended cosinor–based metrics than the actigraphy package and was adopted to comprehensively capture the participants’ circadian rhythms [33].

Although only days with >20 hours of data were included in the analysis, missing values were still present in some datasets. Specifically, for the calculation of peaks, the original program by Rykov et al [17] did not include a mechanism to skip missing values, which could result in errors and prevent the metric from being calculated correctly. To address this limitation, we modified the program by adding a process to skip missing values during the calculation of peaks, ensuring that the metric could be computed accurately using the available data points. In total, we extracted 69 digital biomarkers. The R scripts used for feature extraction are available in Multimedia Appendix 1.

### Statistical Analysis

The normality of the variables was assessed using the Shapiro-Wilk test. For variables following a normal distribution, multiple regression analysis was conducted with the WLM score as the dependent variable, and explanatory variables were selected based on Pearson correlation coefficients. For nonnormally distributed variables, the Spearman correlation analysis was conducted. The Spearman analysis was applied to the entire dataset (all participants) as well as separately to the robust, social prefrailty, and social frailty groups. Kruskal-Wallis tests, followed by Dunn post hoc tests, were used to evaluate differences across the 3 frailty groups. Finally, multinomial logistic regression models were constructed to identify predictors of social frailty status (robust, social prefrailty, and social frailty). On the basis of the results of the Kruskal-Wallis tests, models I and III included age- and sex-adjusted factors as independent variables, whereas models II and IV were unadjusted for these factors. All analyses were conducted using RStudio (version 4.4.1; Posit PBC).

## Results

### Characteristics of the Data and Participants

The average device wearing time per participant was 186 (SD 26) hours, of which an average of 164 (SD 30) hours per participant consisted of complete data recorded on days with at least 20 hours of valid data. Of the 102 participants, only 86 (84.3%) had sufficient data for at least 5 days and were included in the analysis.

The participants had a mean age of 77.14 (SD 5.7; range 65-88) years, and most were women (78/86, 91%). The average daily step count was 7824 (SD 3794), and the mean heart rate was 72.3 (SD 5.9) beats per minute. According to the social frailty criteria defined by Makizako et al [24], the sample was categorized into robust (28/86, 33%), social prefrailty (39/86, 45%), and social frailty (19/86, 22%) groups.

**Figure 2:**
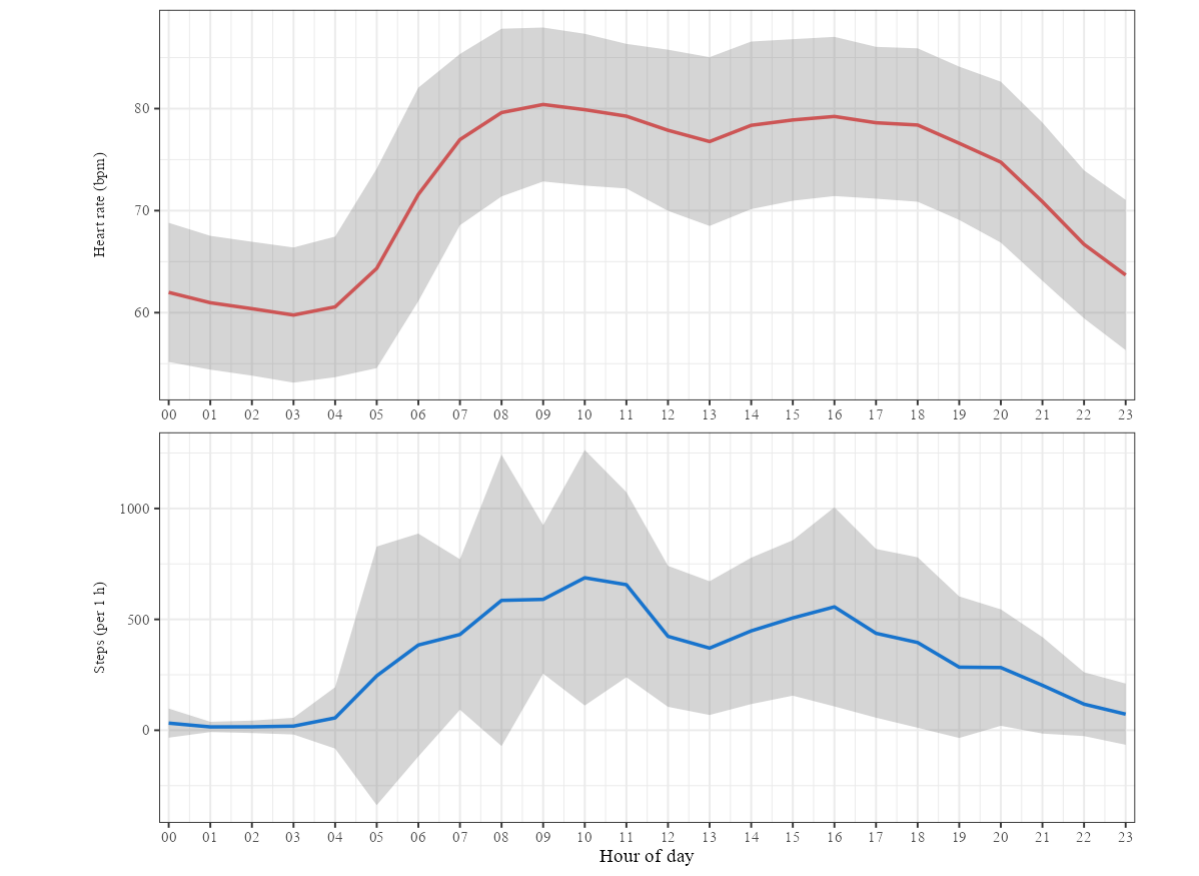
Hourly profiles of heart rate and step count across a 24-hour period among community-dwelling older adults (N=86) in Akita Prefecture, Japan. Data were collected between October 2023 and December 2024 as part of a cross-sectional study examining social frailty and circadian biomarkers. Participants wore Fitbit Inspire 2 and 3 devices for at least 7 consecutive days. The hourly averages (eg, midnight-12:59 AM and 1 AM-1:59 AM) of heart rate and step count were computed using only time windows with at least 30 minutes of valid data per participant. Red and blue lines indicate mean values, and gray shaded areas represent SDs. bpm: beats per minute.

**Figure 3:**
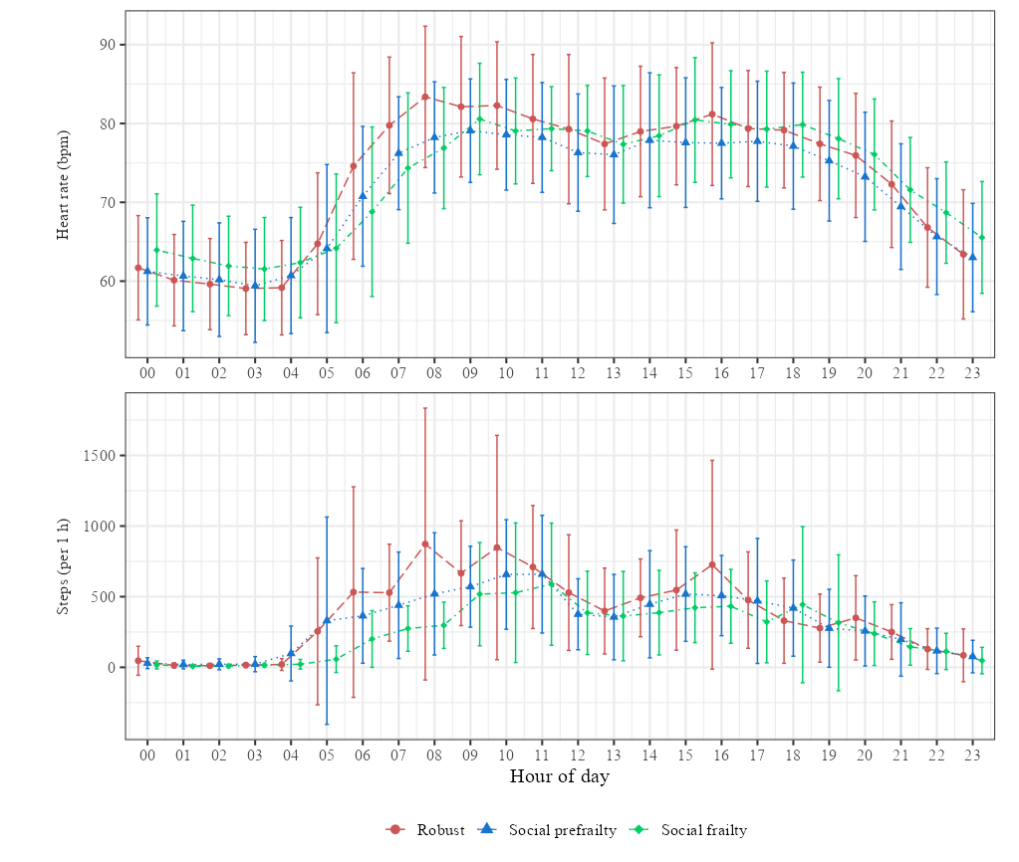
Average 24-hour profiles of heart rate and step count measured using Fitbit Inspire 2 and 3 devices among 3 groups of community-dwelling older adults in Akita Prefecture, Japan: robust (n=28), social prefrailty (n=29), and social frailty (n=19). Data were collected between October 2023 and December 2024 as part of a cross-sectional study on digital biomarkers of social frailty. The hourly means (eg, midnight-12:59 AM and 1 AM-1:59 AM) and SDs were calculated for heart rate and step count. Only time windows with at least 30 minutes of valid data per participant were included in the analysis. The horizontal axis represents the hour of the day. bpm: beats per minute.

Figure 2 presents the averaged 24-hour profiles of hourly heart rate and step count for all participants (n=86). Figure 3 shows the same 24-hour profiles stratified by social frailty status (robust, social prefrailty, and social frailty).

The demographic data for all participants and each social frailty group are summarized in Table 1, including key variables such as age, sex, and variables from physical and cognitive domains, as well as digital biomarkers. Only the key variables are shown in this table, with the full version available in Table S1 in Multimedia Appendix 2.

**Table 1:** Demographic information for all participants and each group (robust, social prefrailty, and social frailty; N=86)^a^.

Variable				All participants		Robust (n=28)		Social prefrailty (n=39)		Social frailty (n=19)
**Demographics**										
	Age (y), mean (SD)		77.14 (5.70)		76.29 (5.38)		77.18 (5.88)		78.32 (6.01)	
	**Sex, n (%)**									
		Female	78 (91)		26 (93)		34 (87)		18 (95)	
		Male	8 (9)		2 (7)		5 (13)		1 (5)	
	Step count, mean (SD)		7824 (3794)		9080 (4529)		7809 (3104)		6005 (3424)	
**Physical domain variables, mean (SD)**										
	Grip strength (kg)		23.81 (5.38)		23.84 (4.37)		24.61 (5.91)		22.11 (5.61)	
	Usual walking speed (m/s)		1.36 (0.29)		1.34 (0.29)		1.38 (0.30)		1.33 (0.30)	
**Cognitive domain variables, mean (SD)**										
	WLM^b^ (score)		12.26 (3.14)		12.62 (3.07)		12.49 (3.26)		11.26 (3.02)	
	TMT-A^c^ (s)		1.33 (0.34)		1.31 (0.39)		1.30 (0.29)		1.40 (0.37)	
	TMT-B^d^ (s)		3.22 (2.80)		3.04 (2.61)		3.42 (3.51)		3.09 (1.13)	
	DSST^e^ (score)		44.70 (13.50)		46.96 (15.81)		45.56 (12.88)		39.58 (10.40)	
**Circadian rhythm metrics—nonparametric analysis, mean (SD)**										
	IS.st^f^		0.46 (0.17)		0.51 (0.19)		0.45 (0.16)		0.38 (0.13)	
	IV.st^g^		1.28 (0.27)		1.20 (0.23)		1.29 (0.27)		1.39 (0.28)	
	L5.st^h^		22 (27)		20 (24)		28 (34)		12 (10)	
	M10.st^i^		575 (287)		655 (339)		570 (230)		465 (292)	
	RA.st^j^		0.92 (0.09)		0.94 (0.06)		0.90 (0.11)		0.93 (0.07)	
	ICV.st^k^		1.12 (0.20)		1.08 (0.25)		1.09 (0.16)		1.25 (0.16)	
**Circadian rhythm metrics—extended cosinor analysis, mean (SD)**										
	Minimum.hr		58.71 (10.04)		58.79 (7.10)		57.19 (12.78)		61.72 (6.82)	
	Amplitude.hr		21.68 (14.24)		21.88 (8.49)		23.57 (19.70)		17.52 (3.97)	
	α parameter.hr		−0.41 (0.24)		−0.48 (0.15)		−0.35 (0.32)		−0.41 (0.13)	
	β parameter.hr		17.59 (20.99)		20.98 (21.19)		13.51 (15.58)		20.96 (30.25)	
	Acrotime.hr (h)		14.01 (1.09)		14.12 (0.85)		13.7 (1.21)		14.50 (1.01)	
	F_pseudo.hr^l^		1562.73 (1361.06)		2011.37 (1314.32)		1483.74 (1553.21)		1063.72 (759.72)	
	UpMesor.hr^m^		6.30 (1.20)		6.15 (0.92)		6.14 (1.41)		6.85 (0.98)	
	DownMesor.hr^n^		21.73 (1.97)		22.08 (1.25)		21.26 (2.55)		22.15 (1.30)	
	MESOR.hr^o^		69.55 (6.07)		69.73 (5.74)		68.97 (6.50)		70.48 (5.98)	
**HR^p^ metrics, mean (SD)**										
	HR (bpm)		72.30 (5.91)		73.10 (5.98)		71.52 (6.00)		72.72 (5.89)	
	SD of the HR (bpm)		12.72 (2.48)		13.71 (2.74)		12.26 (2.25)		12.20 (2.30)	
	RHR^q^ (bpm)		64.04 (6.52)		63.39 (6.57)		63.54 (6.46)		66.01 (6.70)	
	dRHR^r^ (bpm)		8.26 (3.03)		9.71 (3.55)		7.98 (2.69)		6.70 (1.96)	
	RMSSD^s^ of the HR		3.66 (1.03)		3.85 (1.04)		3.59 (1.06)		3.51 (0.99)	
	RMSSD of the HR based on hourly means		7.93 (1.86)		8.53 (2.21)		7.64 (1.62)		7.63 (1.71)	

^a^This table presents demographic characteristics, cognitive performance scores, and rhythm-based digital biomarkers for community-dwelling older adults (N=102) participating in a cross-sectional study conducted in Akita Prefecture, Japan, between October 2023 and December 2024. Participants were classified into 3 groups: robust, social prefrailty, and social frailty. Cognitive tests included the word list memory test, the trail making test versions A and B, and the digit symbol substitution task. Wearable-derived metrics obtained from Fitbit Inspire 2 and 3 devices included average daily step counts and rhythm indicators derived from steps or heart rate data: interdaily stability, intradaily variability, least active 5-hour period, most active 10-hour period, relative amplitude, and interdaily coefficient of variation. The suffix “.st” denotes rhythm indicators based on steps; “.hr” denotes those based on heart rate. Additional physiological measures included resting heart rate, delta resting heart rate, and heart rate variability indexes such as root mean square of successive differences and root mean square of successive differences based on hourly means. Mean 186 (SD 26) sampling hours; mean 164 (SD 30) analyzed hours.

^b^WLM: word list memory.

^c^TMT-A: trail making test version A.

^d^TMT-B: trail making test version B.

^e^DSST: digit symbol substitution task.

^f^IS: interdaily stability.

^g^IV: intradaily variability.

^h^L5: least active 5-hour activity level.

^i^M10: most active 10-hour activity level.

^j^RA: relative amplitude.

^k^ICV: interdaily coefficient of variation.

^l^F_pseudo: the improvement in the fit obtained through the nonlinear estimation of the transformed cosine model.

^m^UpMesor: time of transition from rest to activity.

^n^DownMesor: time of transition from activity to rest.

^o^MESOR: midline estimating statistic of rhythm.

^p^HR: heart rate.

^q^RHR: resting heart rate.

^r^dRHR: delta resting heart rate.

^s^RMSSD: root mean square of successive differences.

### Statistical Analysis Results

**Figure 4:**
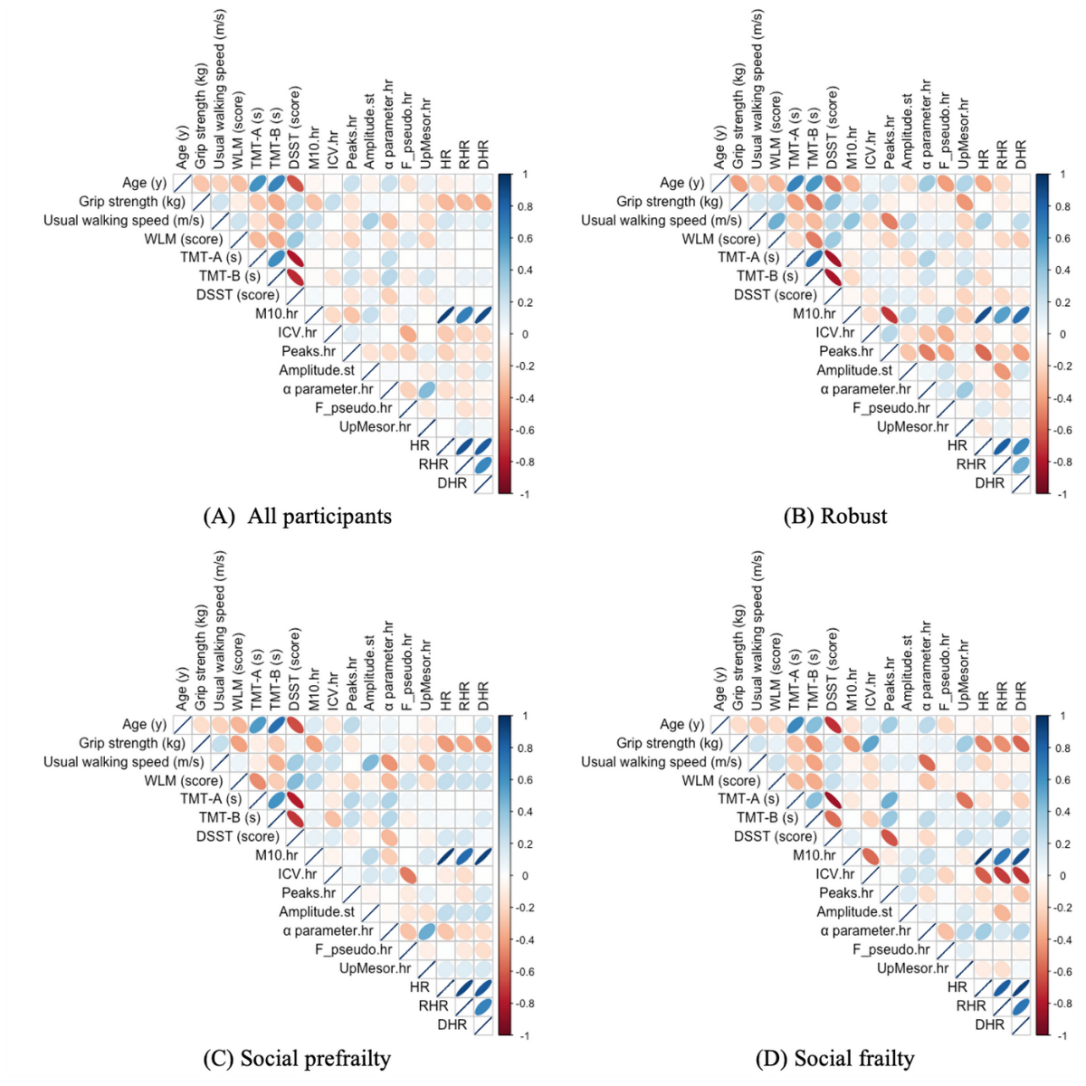
Correlation matrices of rhythm-based digital biomarkers derived from Fitbit Inspire 2 and 3 devices among community-dwelling older adults (N=86) in Akita Prefecture, Japan. This cross-sectional study was conducted between October 2023 and December 2024. Participants were post hoc classified into 4 groups: all participants (A), robust (B; n=28), social prefrailty (C; n=29), and social frailty (D; n=19). The strength and direction of correlations are visualized using ellipses—deep red indicates strong negative correlations, deep blue indicates strong positive correlations, and lighter shades indicate weaker correlations. Variables other than age, physical function, and cognitive function are displayed only if their correlation coefficients showed significant differences across at least 3 of the 4 panels. α parameter.hr: the width of troughs and peaks in the rhythm; Amplitude.hr: half the extent of predictable variation within a cycle; DHR: daytime heart rate (average heart rate between 2 PM and 4 PM); dRHR: delta resting heart rate (HR); DSST: digit symbol substitution task; F_pseudo.hr: the improvement in the fit obtained through the nonlinear estimation of the transformed cosine model derived from HR; ICV.hr: intradaily coefficient of variation; M10.hr: most active 10-hour activity level; Peaks.hr: number of heart rate peaks per day detected in time-series data; RHR: resting HR; TMT-A: Trail Making Test version A; TMT-B: Trail Making Test version B; UpMesor.hr: time of transition from rest to activity; WLM: word list memory.

Figure 4 illustrates the results of the Spearman correlation analysis conducted across the entire dataset (all participants) and separately for the robust, social prefrailty, and social frailty groups. Age, physical domain, and cognitive domain variables are included in the analysis, whereas other variables are reported only if their correlation coefficients showed significant associations in at least 3 of the panels. Tables S2-S5 in Multimedia Appendix 2 provide detailed correlation coefficients.

Table 2 presents the results of the multiple linear regression analyses with WLM score as the dependent variable. In total, 2 models were analyzed. Model I included age and ICV × 100 as independent variables, whereas model II added one more variable to the predictors. ICV × 100 represents the step-based intrahourly CV scaled by multiplying the original values by 100 to enhance interpretability.

**Table 2:** Models according to multiple logistic regression analysis^a^.

			β coefficient (SE; 95% CI)		*P* value
**Model I^b^**					
	Age	−0.15 (0.06; –0.26 to –0.03)		.01	
	ICV.st^c^ × 100	−0.04 (0.02; –0.07 to –0.01)		.02	
**Model II^d^**					
	Age	−0.13 (0.06; –0.25 to –0.02)		.03	
	ICV.st × 100	−0.04 (0.02; –0.07 to 0.00)		.03	
	Peaks.hr^e^	−0.39 (0.29; –0.97 to 0.20)		.19	

^a^The dependent variable for the analysis was word list memory score, derived from cognitive assessments. Independent variables included age, the step-based intrahourly coefficient of variation standardized and scaled by 100, and the number of heart rate peaks per day detected in time-series data. This cross-sectional study was conducted among community-dwelling older adults in Akita Prefecture, Japan, with data collected between October 2023 and December 2024. Model fit was evaluated using *R*^2^ (proportion of variance explained), adjusted *R*^2^ (adjusted for the number of predictors), and the *F* statistic (testing the overall model significance).

^b^*R*^2^=0.150; adjusted *R*^2^=0.129; *F* statistic *P*=.001.

^c^ICV.st: step-based intradaily coefficient of variation.

^d^*R*^2^=0.167; adjusted *R*^2^=0.137; *F* statistic *P*=.002.

^e^Peaks.hr: number of heart rate peaks per day detected in time-series data.

In model I, both age (β=−0.15, 95% CI −0.26 to −0.03; *P*=.01) and ICV × 100 (β=−0.04, 95% CI −0.07 to −0.01; *P*=.02) were significant predictors of WLM score. In model II, age (β=−0.13, 95% CI −0.25 to −0.02; *P*=.03) and ICV × 100 (β=−0.04, 95% CI −0.07 to 0.00; *P*=.03) remained significant.

Table 3 summarizes the results of the Kruskal-Wallis and Dunn post hoc tests for digital biomarkers across the robust, social prefrailty, and social frailty groups. The Dunn post hoc tests were conducted following the Kruskal-Wallis test to identify pairwise differences, and the *P* values from these tests were adjusted using the Bonferroni method. The table reports only the 17 variables with significant associations identified through the Dunn test (*P*<.05). Notably, comparisons between the robust and social frailty groups revealed significant differences in 13 biomarkers.

**Table 3:** Analysis of digital biomarkers by social frailty categories using the Kruskal-Wallis and Dunn tests^a^.

Variable	Robust (n=28), median (IQR)	Social prefrailty (n=39), median (IQR)	Social frailty (n=19), median (IQR)	Overall *P* value	Robust vs social prefrailty *P* value	Robust vs social frailty *P* value	Social prefrailty vs social frailty *P* value
Steps	7685 (6562)	7488 (4832)	5114 (2782)	.02	.58	.008	.04
IS.st^b^	0.52 (0.21)	0.43 (0.18)	0.38 (0.19)	.03	.15	.01	.28
IV.st^c^	1.22 (0.23)	1.25 (0.41)	1.33 (0.42)	.10	.50	.045	.22
M10.st^d^	577 (406)	527 (342)	383 (261)	.06	.78	.03	.09
L5.st^e^	10 (16)	16 (19)	10 (10)	.07	.20	.63	.04
ICV.st^f^	1.04 (0.24)	1.08 (0.22)	1.24 (0.14)	.001	.61	<.001	.003
AC.st.60m^g^	0.30 (0.17)	0.30 (0.16)	0.18 (0.21)	.07	>.99	.046	.08
AC.st.30m^h^	0.24 (0.17)	0.25 (0.16)	0.14 (0.13)	.05	.66	.02	.09
AC.st.15m^i^	0.18 (0.15)	0.18 (0.16)	0.11 (0.10)	.08	.63	.04	.16
F_pseudo.st^j^	111.30 (169.36)	65.50 (150.43)	45.74 (74.92)	.03	.30	.01	.14
SD of the HR^k^ (bpm)	13.28 (2.74)	11.95 (2.79)	11.74 (2.32)	.03	.03	.047	>.99
dRHR^l^ (bpm)	10.23 (5.02)	7.31 (3.27)	6.85 (2.86)	.008	.07	.003	.22
α parameter.hr	−0.49 (0.17)	−0.36 (0.19)	−0.36 (0.16)	.04	.02	.11	>.99
F_pseudo.hr^m^	1586.53 (1436.61)	1036.27 (1448.31)	665.80 (1064.46)	.02	.04	.02	.81
UpMesor.hr^n^	6.39 (1.13)	6.39 (1.15)	6.87 (0.95)	.046	.87	.03	.06

^a^Comparison of digital biomarkers related to circadian rhythm and heart rate among the robust, social prefrailty, and social frailty groups using the Kruskal-Wallis and post hoc Dunn tests. Data were collected in a cross-sectional study conducted between October 2023 and December 2024 among community-dwelling older adults in Akita Prefecture, Japan. The table reports the median and IQR for each variable by group. *P* values reflect overall group differences and pairwise comparisons between groups, with significance corrected using the Bonferroni method. Only variables showing at least one significant pairwise difference (*P*<.05) are included in the table. The suffix “.st” denotes rhythm indicators based on steps; “.hr” denotes those based on heart rate.

^b^IS: interdaily stability.

^c^IV: intradaily variability.

^d^M10: most active 10-hour activity level.

^e^L5: least active 5-hour activity level.

^f^ICV: intradaily coefficient of variation.

^g^AC.st.60m: autocorrelation of step counts aggregated in 60-minute intervals.

^h^AC.st.30m: autocorrelation of step counts aggregated in 30-minute intervals.

^i^AC.st.15m: autocorrelation of step counts aggregated in 15-minute intervals.

^j^F_pseudo.st: the improvement in the fit obtained through the nonlinear estimation of the transformed cosine model derived from steps.

^k^HR: heart rate.

^l^dRHR: delta resting heart rate.

^m^F_pseudo.hr: the improvement in the fit obtained through the nonlinear estimation of the transformed cosine model derived from heart rate.

^n^UpMesor: time of transition from rest to activity.

Table 4 summarizes the results of the multiple logistic regression analysis, with models I and III adjusted for age and sex and models II and IV excluding these adjustments. The robust group was used as the reference category.

**Table 4:** Models according to multiple logistic regression analysis^a^.

				β coefficient (SE)		Odds ratio (95% CI)		*P* value
**Model I^b^**								
	**Social prefrailty group**							
		Age (y)	0.01 (0.03)		1.01 (0.95-1.08)		.76	
		Sex (male=1; female=0)	−0.53 (0.76)		0.59 (0.13-2.58)		.49	
		dRHR^c^	−0.21 (0.10)		0.82 (0.68-0.99)		.04	
		α parameter × 100	0.05 (0.02)		1.05 (1.01-1.08)		.006	
		UpMesor.hr^d^	−0.55 (0.25)		0.58 (0.36-0.93)		.03	
		F_pseudo.hr^e^	0.00 (0.00)		1.00 (0.99-1.00)		.93	
	**Social frailty group**							
		Age (y)	0.06 (0.03)		1.06 (0.99-1.13)		.08	
		Sex (male=1; female=0)	−1.98 (0.38)		0.14 (0.07-0.28)		<.001	
		dRHR	−0.37 (0.15)		0.69 (0.50-0.95)		.01	
		α parameter × 100	0.01 (0.02)		1.01 (0.97-1.05)		.61	
		UpMesor.hr	0.33 (0.30)		1.39 (0.77-2.50)		.27	
		F_pseudo.hr	0.00 (0.00)		1.00 (0.99-1.00)		.17	
**Model II^f^**								
	**Social prefrailty group**							
		dRHR	−0.20 (0.09)		0.82 (0.68-0.97)		.02	
		α parameter.hr × 100	0.05 (0.01)		1.05 (1.02-1.07)		<.001	
		UpMesor.hr	−0.56 (0.11)		0.57 (0.46-0.71)		<.001	
		F_pseudo.hr	−0 (0.00)		1.00 (1.00-1.00)		.97	
	**Social frailty group**							
		dRHR	−0.31 (0.13)		0.74 (0.58-0.94)		.02	
		α parameter.hr × 100	0.01 (0.02)		1.01 (0.98-1.04)		.68	
		UpMesor.hr	0.37 (0.15)		1.45 (1.09-1.94)		.01	
		F_pseudo.hr	0.00 (0.00)		1.00 (1.00-1.00)		.19	
**Model III^g^**								
	**Social prefrailty group**							
		Age (y)	0.01 (0.05)		1.01 (0.92-1.11)		.79	
		Sex (male=1; female=0)	0.02 (0.05)		1.02 (0.92-1.13)		.76	
		ICV.st^h^ × 100	0.00 (0.02)		1.00 (0.97-1.03)		.96	
		α parameter.hr × 100	0.05 (0.02)		1.05 (1.01-1.12)		.02	
		UpMesor.hr	−0.59 (0.31)		0.55 (0.30-1.01)		.06	
		SD of the HR^i^	−0.29 (0.12)		0.75 (0.59-0.96)		.02	
	**Social frailty group**							
		Age (y)	0.04 (0.06)		1.04 (0.93-1.16)		.47	
		Sex (male=1; female=0)	−2.22 (1.65)		0.11 (0.01-1.69)		.18	
		ICV.st × 100	0.05 (0.02)		1.05 (1.01-1.11)		.01	
		α parameter.hr × 100	0.01 (0.02)		1.01 (0.96-1.07)		.68	
		UpMesor.hr	0.44 (0.39)		1.55 (0.72-3.35)		.26	
		SD of the HR	−0.18 (0.15)		0.83 (0.63-1.11)		.20	
**Model IV^j^**								
	**Social prefrailty group**							
		ICV.st × 100	0.00 (0.01)		1.00 (0.97-1.03)		.99	
		α parameter.hr × 100	0.05 (0.02)		1.05 (1.01-1.09)		.02	
		UpMesor.hr	−0.60 (0.31)		0.55 (0.30-1.01)		.06	
		SD of the HR	−0.29 (0.12)		0.75 (0.59-0.94)		.02	
	**Social frailty group**							
		ICV.st × 100	0.04 (0.02)		1.04 (1.01-1.08)		.01	
		α parameter.hr × 100	0.01 (0.02)		1.01 (0.96-1.06)		.70	
		UpMesor.hr	0.46 (0.38)		1.58 (0.74-3.32)		.24	
		SD of the HR	−0.17 (0.14)		0.85 (0.64-1.12)		.25	

^a^Multiple logistic regression analyses predicting social prefrailty and social frailty status relative to the robust group among community-dwelling older adults in Akita Prefecture, Japan. Data were collected between October 2023 and December 2024 in a cross-sectional study. This table summarizes the results of 4 models (models I-IV), each using a different combination of predictors related to heart rate variability and rhythm metrics derived from wearable Fitbit devices. The dependent variable was group classification (robust, social prefrailty, or social frailty), with the robust group used as the reference category. Independent variables included delta resting heart rate, α parameter (scaled by 100), rest-activity transition timing, step-based intradaily variability, model fit from the extended cosinor analysis, and SD of the heart rate. The suffix “.st” denotes rhythm indicators based on steps; “.hr” denotes those based on heart rate. Model fit was evaluated using the likelihood-ratio test, the Nagelkerke *R*^2^, and the Akaike information criterion (AIC). Model IV demonstrated the best fit, indicated by the lowest AIC (170.1) and a relatively high *R*^2^ (0.351).

^b^Likelihood-ratio test *P* value=.002; Nagelkerke *R*^2^=0.343; AIC=179.0.

^c^dRHR: delta resting heart rate.

^d^UpMesor: time of transition from rest to activity.

^e^F_pseudo.hr: the improvement in the fit obtained through the nonlinear estimation of the transformed cosine model derived from heart rate.

^f^Likelihood-ratio test *P*<.001; Nagelkerke *R*^2^=0.315; Akaike information criterion=173.9.

^g^Likelihood-ratio test *P*=.001; Nagelkerke *R*^2^=0.378; Akaike information criterion=175.2.

^h^ICV: intradaily coefficient of variation.

^i^HR: heart rate.

^j^Likelihood-ratio test *P*<.001; Nagelkerke *R*^2^=0.351; Akaike information criterion=170.1.

In model II, the dRHR showed significant negative associations with both social prefrailty (OR 0.82, 95% CI 0.68-0.97; *P*=.02) and social frailty (OR 0.74, 95% CI 0.58-0.94; *P*=.02). Similarly, UpMesor.hr demonstrated significant associations with both social prefrailty (OR 0.57, 95% CI 0.46-0.71; *P*<.001) and social frailty (OR 1.45, 95% CI 1.09-1.94; *P*=.01).

In model IV, the α parameter.hr × 100 was significantly associated with social prefrailty (OR 1.05, 95% CI 1.01-1.09; *P*=.02). The SD of the heart rate showed a significant negative association with social prefrailty (OR 0.75, 95% CI 0.59-0.94; *P*=.02). For social frailty, ICV.st × 100 demonstrated a significant positive association (OR 1.04, 95% CI 1.01-1.08; *P*=.01).

The results of models I and III, which were adjusted for age and sex, were generally consistent with their corresponding models (model I corresponds to models II, and model III corresponds to model IV). The primary difference was observed in UpMesor.hr, which was significantly associated with both social prefrailty and social frailty in model II but only with social prefrailty in model I. Regarding other variables, dRHR, ICV.st × 100, the α parameter.hr × 100, and the SD of the heart rate showed consistent associations across corresponding models.

## Discussion

### Principal Findings

Our results lent support to the hypothesis that the frailty group would exhibit significant associations with RAR parameters (nonparametric and extended cosinor analysis) and heart rate–based parameters. The observed significant relationships with dRHR, UpMesor, and other heart rate–derived metrics validate their relevance in understanding social frailty. In addition, Fitbit-derived RAR metrics were found to be associated with cognitive function, particularly memory decline, in partial alignment with the proposed hypothesis.

The results of the analyses using the multinomial logistic regression models partially supported the primary hypothesis. Among the step-based rhythm metrics, only the ICV was significantly associated with social prefrailty. On the other hand, the dRHR and UpMesor showed significant associations with both social frailty and prefrailty. Furthermore, the α parameter and the SD of the heart rate were significant predictors of social prefrailty. Notably, except for the ICV, all the significant predictors of social frailty were derived from heart rate data.

First, the dRHR showed significant negative associations with both social prefrailty (OR 0.82, 95% CI 0.68-0.97; *P*=.02) and social frailty (OR 0.74, 95% CI 0.58-0.94; *P*=.02) in model II. The dRHR reflects the difference between the daily average heart rate and the RHR, which may provide insights into the activity levels and physiological responses to rest and activity. In this study, we found that the dRHR values were smaller in the social frailty and prefrailty groups, potentially indicating reduced daytime activity levels and a closer approximation of the average heart rate to the RHR.

Figure 2 illustrates our finding of lower daytime heart rates and higher nocturnal heart rates in the social frailty group. This pattern corresponds to smaller dRHR values, suggesting a possible disruption in the typical fluctuations between rest and activity. Notably, insufficient nocturnal heart rate reduction in persons with social frailty might contribute to an elevated RHR. In addition, increased physical activity has been associated with reduced RHRs, likely mediated by autonomic nervous system regulation [34]. Therefore, lower dRHR values might reflect both reduced daytime activity and limited heart rate reduction during rest. In this context, dRHR can be interpreted as a metric associated with variations in heart rate across periods of activity and rest.

Studies on the association between the severity of depression and heart rate variation have suggested that reduced activity levels can lead to altered heart rate fluctuation patterns [35]. Specifically, a higher nocturnal resting mean heart rate has been associated with more severe depressive symptoms (β=0.09; *P*<.001), whereas greater daytime heart rate variation (β=−0.34; *P*<.001) and a higher SD of daytime heart rate (β=−0.18; *P*<.001) have been linked to a lower severity of depression [35].

Similarly, previous research has identified a high RHR as a risk factor for dementia [36], worsening of depression [35], noncardiac disability [37], and physical frailty [38]. Social frailty has also been reported to predict disability [24], cognitive decline [39], depression [40], and physical frailty [6,39,41]. On the basis of these findings, dRHR may function as a comprehensive indicator reflecting these interconnected factors.

However, research on the dRHR is limited. Only the studies by Rykov et al [16,17] have examined dRHR in patients with heart disease and depression. Our study is the first to report on dRHR in the context of social frailty and has highlighted its relevance and potential value for understanding social frailty.

In addition, another heart rate indicator, the SD of the heart rate, was identified as a predictor of social prefrailty (OR 0.75, 95% CI 0.59-0.94; *P*=.02; model IV). Similarly to dRHR, the SD of the heart rate can be interpreted as an indicator of the heart rate fluctuations associated with daily physical activity levels. It is also classified as a measure of the HRV, a widely recognized method for assessing autonomic nervous system function. The SD of the heart rate has been classified as a time-domain HRV metric in previous research [42]. While the SD of the heart rate has been less frequently studied compared with other HRV metrics such as the RMSSD and SD of the NN interval, its potential relevance in the context of social frailty is noteworthy. In this study, we also calculated the RMSSD, but no significant differences were observed between groups. While HRV is a well-established metric for assessing autonomic nervous system function, its specific significance in social frailty remains underexplored. Future research should incorporate not only time-domain measures such as RMSSD, which primarily reflect parasympathetic nervous system activity, but also frequency-domain metrics, including low- and high-frequency components and their ratio (LF/HF), which reflect the balance between sympathetic and parasympathetic activity [43]. This may lead to a deeper understanding of the role of HRV in the context of social frailty.

Second, this study found that the UpMesor was delayed in the social frailty group (OR 1.45, 95% CI 1.09-1.94; *P*=.01; model II). Although previous studies have not used heart rate–based UpMesor, our results align with reports from previous studies that delayed activity onset is associated with depressive symptoms and functional decline [44]. Given the association between social frailty and depression [45-47], delayed activity rhythms may represent a characteristic feature of social frailty.

Conversely, the UpMesor was advanced in the social prefrailty group (OR 0.57, 95% CI 0.46-0.71; *P*<.001; model II). Typically, social frailty progresses linearly from robust to prefrailty and then to frailty, suggesting that UpMesor would also be progressively delayed across these stages. However, our findings present an apparent contradiction.

According to the report by Ji et al [48], UpMesor, DownMesor, and acrophase are parameters reflecting temporal rhythms, including the onset and cessation of nighttime sleep and peak daytime activity levels. The observed opposing patterns of UpMesor suggest the importance of understanding how specific temporal rhythm elements impact health across the stages of social frailty.

Previous research has reported on acrophase, revealing complex associations. For example, delayed acrophase has been linked to developing dementia or mild cognitive impairment [49], whereas early acrophase has been associated with cognitive decline [50,51]. These studies indicate that temporal rhythms do not exhibit simple linear relationships with health; both excessively delayed and advanced rhythms may negatively impact health.

The observed delay in UpMesor in the social frailty group and its advancement in the social prefrailty group may support this nonlinear association. Specifically, early activity onset in the prefrailty stage and delayed activity onset in the frailty stage may each contribute to health risks. This finding underscores the need for further research to examine how factors such as sex, age, and physiological characteristics might influence these associations.

Third, the α parameter × 100 was significantly associated with social prefrailty (OR 1.05, 95% CI 1.01-1.09; *P*=.02; model IV), suggesting that an increase in α may heighten the risk of social prefrailty. High α values reflect shorter activity periods and longer rest periods, corresponding to insufficient daytime activity. This aligns with the findings of Smagula et al [44], which linked high α values to increased health risks such as depression.

However, α may also indicate rhythm clarity, as suggested by Ji et al [48] using the sleep regularity, satisfaction, alertness, timing, efficiency, and duration model [48]. Therefore, α must be interpreted cautiously as it may reflect both the quantity and quality of daytime activity and nighttime rest.

Fourth, the ICV × 100 was significantly associated with social frailty (OR 1.04, 95% CI 1.01-1.08; *P*=.01; model IV). ICV reflects the intrahourly CV, with higher values indicating greater variability in activity levels. In this study, individuals with social frailty exhibited more frequent alternations between short activity bursts and rest, consistent with the report that reduced physical activity and limited social engagement are associated with the risk of frailty [6]. These results suggest that intradaily rhythm instability may be a key feature of social frailty.

In addition, we found a significant association between ICV and WLM score (β=−0.04; *P*=.02), suggesting that higher rhythm variability may be linked to lower cognitive performance. This finding aligns with those of previous research indicating that increased IV and decreased IS are associated with cognitive decline [52,53]. These results may imply that the ICV metric, which reflects short-term rhythm fragmentation, is related to cognitive function. However, as Fitbit does not record activity counts, relying solely on step-based data may lead to underestimation of activity levels in individuals with impaired mobility.

### Strengths and Limitations

One of the significant strengths of this study is its use of Fitbit data to elucidate new rhythm-related characteristics associated with social frailty. Fitbit devices have demonstrated acceptable precision comparable to that of research-grade devices and are also affordable and accessible, making them promising tools for the development of digital biomarkers. In this study, step count and heart rate data recorded by Fitbit were used to replicate rhythm metrics based on the scripts by Rykov et al [17], and new rhythm metrics were also calculated. We demonstrated in this study that this comprehensive approach offers the potential for evaluating rhythm-related characteristics in social frailty.

Furthermore, the combined use of nonparametric metrics and extended cosinor metrics has shown that multifaceted evaluation of rhythm characteristics is achievable. Nonparametric metrics are well suited for capturing the daily regularity of rhythms, whereas extended cosinor metrics are useful for analyzing phase changes along the time axis. This complementary use uncovered new aspects of social frailty that were not previously identifiable through conventional methods.

In addition, extended cosinor metrics, which have traditionally been calculated based on activity counts, were applied to heart rate data in this study, revealing new possibilities for rhythm analysis. This development suggests that rhythm evaluations can be conducted even for individuals with reduced walking ability or limited daytime movement. The use of heart rate–based data expands the scope of analysis to groups that have been challenging to evaluate previously, providing a more comprehensive understanding of the rhythm characteristics.

Despite its strengths, this study had several limitations. First, the sample size posed a constraint. Due to the limited sample size, the findings should be interpreted as observational results. For example, determining the “optimal range” for UpMesor requires validation in larger sample sizes. Furthermore, the small sample size restricted our ability to conduct comprehensive analyses integrating nonlinear models or multiple health outcomes.

Second, sex differences may have influenced the results. The potential impact of sex on the outcomes necessitates cautious interpretation of the regression model estimates. Future research should incorporate sex considerations to provide more nuanced insights.

Third, there is the issue of uncertainty surrounding the standardization of social frailty indexes. According to a meta-analysis conducted by Jia et al [54], lack of a standardized social frailty index and reliance on operational choices by individual researchers remain significant challenges. This calls for further discussion and validation of the reliability of social frailty indexes.

Fourth, although a significant association between rhythm metrics and cognitive function (WLM score) was observed, this analysis was exploratory and secondary in nature. As the primary objective of this study was to model social frailty, these findings should be interpreted as preliminary. Advanced modeling techniques such as machine learning were not used in this study due to the limited sample size and the descriptive aim of the cognitive analysis. Future studies with larger samples should incorporate machine learning to develop predictive models for cognitive outcomes and validate the utility of rhythm-based digital biomarkers.

In addition, the interpretation of extended cosinor metrics presents a challenge. The current body of research on extended cosinor metrics remains limited, and careful consideration tailored to specific objectives and populations is required. Unlike nonparametric metrics, cosinor metrics do not allow for simple binary evaluations such as “stable” or “unstable.” Both excessive stability and instability of rhythms may be associated with health risks, which necessitates a comprehensive approach to rhythm evaluation.

Finally, there are limitations related to Fitbit data. While Fitbit and similar smartwatches provide minute-by-minute step data through their application programming interfaces, they do not provide activity count data. This lack of activity count data raises questions about the validity of rhythm metrics derived solely from step data, highlighting the need for future research to address this issue.

### Conclusions

This study revealed associations between novel rhythm metrics derived from step count and heart rate data recorded via Fitbit devices and social frailty. These findings suggest that digital biomarkers for evaluating social frailty and its risk factors can be derived from data provided by consumer-grade wearable devices, which are affordable and accessible.

In particular, it was shown that heart rate–based metrics such as dRHR, UpMesor, and the α parameter may play a crucial role in the assessment of social frailty and prefrailty. These metrics have the potential to complement existing activity count–based rhythm analyses by capturing new dimensions of health that were previously overlooked.

Furthermore, the findings of our study suggest that social frailty is associated with specific rhythm characteristics such as decreased daytime activity levels and increased nighttime heart rates. These findings indicate that rhythm metrics may be valuable for early detection and assessment of social frailty.

This research provides preliminary insights into the evaluation of social frailty using Fitbit data. However, limitations such as the small sample size and uncertainty surrounding standardized indexes for social frailty must be addressed. Future studies should focus on validating these findings in larger samples and conducting comparative research using other devices. In addition, further exploration is needed to determine whether heart rate–based metrics can be applied to assess other health conditions and diseases.

## Appendix

Multimedia Appendix 1 R-code scripts for data processing.

Multimedia Appendix 2 Detailed statistical analysis tables.

## Abbreviations

CV: coefficient of variation
DownMesor: time of transition from activity to rest
dRHR: delta resting heart rate
HRV: heart rate variability
ICV: intradaily coefficient of variation
IS: interdaily stability
IV: intradaily variability
L5: least active 5-hour activity level
M10: most active 10-hour activity level
MESOR: midline estimating statistic of rhythm
OR: odds ratio
RA: relative amplitude
RAR: rest-activity rhythm
RHR: resting heart rate
RMSSD: root mean square of successive differences
UpMesor: time of transition from rest to activity
WLM: word list memory

## Data Availability

The datasets generated during the current study are not publicly available due to ethical restrictions but may be available from the corresponding author upon reasonable request and approval by the institutional review board.
